# The global prevalence of peripheral neuropathy following chemotherapy in cancer patients: a systematic review and meta-analysis

**DOI:** 10.1186/s13023-026-04256-y

**Published:** 2026-02-06

**Authors:** Nader Salari, Atefeh Galehdari Fard, Amir Abdolmaleki, Hadis Mosafer, Shamarina Shohaimi, Masoud Mohammadi

**Affiliations:** 1 Sleep Disorders Research Center, Kermanshah University of Medical Sciences, Kermanshah, Iran; 2 Department of Biostatistics, School of Health, Kermanshah University of Medical Sciences, Kermanshah, Iran; 3 Department of Nursing, Faculty of Nursing and Midwifery, Urmia University of Medical Sciences, Urmia, Iran; 4 Department of Operating Room, Nahavand School of Allied Medical Sciences, Hamadan University of Medical Sciences, Hamadan, Iran; 5 Student Research Committee, Kermanshah University of Medical Sciences, Kermanshah, Iran; 6 Department of Biology, Faculty of Science, University Putra Malaysia, Serdang, Selangor Malaysia; 7 Research Center for Social Determinants of Health, Jahrom University of Medical Sciences, Jahrom, Iran

**Keywords:** Chemotherapy, Peripheral, Neuropathy, Peripheral neuropathy, Peripheral nervous system disease

## Abstract

**Background:**

Chemotherapy-induced peripheral neuropathy (CIPN) is a major cause of dose reduction, drug modification, or drug discontinuation in cancer patients which negatively impacts the overall well-being of cancer patients and medication procedures. This systematic review and meta-analysis investigation aimed to determine the global prevalence of CIPN in cancer patients.

**Methods:**

Various scientific databases (PubMed, Scopus, Web of Science, Embase, ScienceDirect, and Google Scholar) were systematically searched (by July 2023) for published studies reporting the CIPN prevalence. Meta-analysis was applied based on the Random Effect model and subgrouping was considered using the CIPN scales. Also, the heterogeneity was assessed based on the I^2^ index.

**Results:**

Following the assessment of 49 eligible studies (n:33,667 participants), the overall CIPN prevalence was reported 51.9% (95% CI: 45-58.7). According to the Composite Scales tool, the highest CIPN prevalence was 69.6% (95%CI: 50–84).

**Conclusion:**

The prevalence of CIPN in cancer patients was found at a high level. According to the high number of cancer survivors, the integration of necessary clinical strategies for screening, prevention, and treatment of CIPN into consistent clinical guidelines is strictly recommended. Probably these guidelines can reduce the CIPN occurrence and cancer treatment costs.

**Clinical trial number:**

Not applicable.

## Background

Cancer is a considerable health issue, globally [1]. Approximately 19 to 20 million people are diagnosed with cancer each year [2]. According to updated estimates from the International Agency for Research on Cancer (IARC), approximately one in five men or women will develop cancer in their lifetime in 2022, while about one in nine men and one in 12 women will die from it [2]. Lung cancer was the most common cancer diagnosed in 2022, accounting for approximately 2.5 million new cases, or one in eight, worldwide, followed by female breast, colorectal, prostate and stomach cancers [2]. Breast and lung cancer were the most common cancers in women and men, respectively, according to the report. Based on demographic projections, the number of new cancer cases is expected to reach 35 million by 2050 [2].

The process of chemotherapy is a common systematic treatment for cancer patients which leads to Chemotherapy-induced peripheral neuropathy (CIPN) occurrence in cancer survivors [3]. Chemotherapy-induced peripheral neuropathy (CIPN) is a common dose-limiting side effect experienced by patients undergoing cancer treatment [3, 4]. Approximately 30 to 40% of patients undergoing neurotoxic chemotherapy develop CIPN. Chemotherapy-induced peripheral neuropathy is a common side effect of several classes of chemotherapy drugs, including platinum-based drugs, taxanes, vinca alkaloids, bortezomib, and thalidomide analogs [3, 4]. These drugs can cause nerve damage, leading to symptoms such as tingling, numbness, pain, and temperature sensitivity in the hands and feet. While the exact mechanisms are not fully understood, it is believed that these drugs can directly damage nerve fibers, cause inflammation, and disrupt nerve signaling pathways [3, 4]. The condition is often associated with predominantly sensory pain and can lead to long-term complications in survivors [3, 4]. Although the CIPN refers to dysfunction of sensory, motor, and autonomic neurons, mostly the CIPN manifests as sensory impairment. Common symptoms of CIPN include paresthesia, hypoesthesia, neuropathic sensations, causalgia, shooting pains, or electric shock-like sensations [3, 4].

Although surgery is a viable treatment option for various cancers, the recurrence rate is also a consideration in these cancers, prompting the use of additional treatments to improve outcomes in the treatment of multiple cancers [5]. Adjuvant and preoperative chemotherapy is performed to eradicate primary micrometastatic disease, reducing the recurrence rate and improving survival outcomes [5]. Accordingly, the addition of targeted therapies to chemotherapy has improved the response rate and tumor resectability when administered preoperatively [5].CIPN disrupts daily activities and function, decreases life quality, and impacts the overall well-being of cancer patients, negatively [6]. It is a major cause of dose reduction, modification, or discontinuation of drugs by the patients which potentially impacts therapeutic effects of medications [3]. 37–84% of the patients experience the CIPN three months after treatment termination. This statistic highlights the significant impact of long-lasting neuropathy and unavailability of sufficient preventive or treatment options for CIPN [7].

According to the importance of CIPN and the associated detrimental effects on survival, quality of life, and well-being of cancer survivors, various studies have been conducted to determine the prevalence of CIPN in different populations [8–10]. Measurement of CIPN with various and numerous tools can potentially cause different reports of CIPN prevalence [7]. The lack of comprehensive and up-to-date meta-analyses regarding the prevalence of CIPN hinders the researchers and policymakers from generating informed decisions and developing effective interventions for management of this important issue. Seretny et al. (2014) conducted a systematic review and meta-analysis investigation reporting the prevalence, incidence, and prediction of CIPN-associated factors [11]. In Seretny`s study, 30 included papers reporting the rate of CIPN incidence, were selected from the prospective cohort and RCT studies. Considering that more than 10 years have passed since Seretny’s study and the lack of a comprehensive and updated meta-analysis, this meta-analysis study was designed to estimate the global prevalence of CIPN in cancer patients.

## Methods

This systematic review and meta-analysis study was conducted based on the PRISMA expanded guidelines [12].

### Data sources and searching strategy

The searching strategy was applied using electronic databases of PubMed, Web of Science, Scopus, ScienceDirect, Embase, and Google Scholar. All searching processes were conducted by July 2023 using main keywords selected based on the PICOT framework, including “*Cancer*”, “*Chemotherapy*”, “*Peripheral Neuropathy*”, and “*Epidemiology*”. All the keywords were identified based on the Medical Subject Headings (MeSH), EMTREE terms, and other routine available synonyms. Following the searching strategy implementation, all gathered papers were imported into Citation Management Software (EndNote. x8).

### Eligibility of criteria

All descriptive cross-sectional, correlational cross-sectional, and survey studies reporting the prevalence of CIPN in cancer patients were included for data analysis. Whole papers were selected in English. Besides, all studies reporting the prevalence of peripheral neuropathy (PN) resulted from non-chemotherapy factors (such as HIV/AIDS, diabetes, neurological diseases, and autoimmune diseases), case reports, case series, cohorts, longitudinal investigations, case-control studies, experimental assessments, review studies, and secondary analyses were totally excluded from the investigation.

### Study selection

According to screening process, duplicate investigations were removed following paper selection. Subsequently, initial screening of articles was applied based on the “Title” and “Abstract”. Then, full-text of remaining articles was evaluated in secondary screening process. Both stages of screening (primary and secondary) were evaluated based on the Inclusion/Exclusion criteria and irrelevant studies were ignored for more assessment. For probable bias prevention, all stages of data source reviewing and data extraction were totally conducted by two independent researchers. Finally, a third author was responsible for management in case of any disagreements.

### Paper quality appraisal

The Cochrane Collaboration recommends using the Newcastle-Ottawa Scale (NOS) to assess the rate of bias risk in observational studies. NOS evaluates selection, comparability, and outcome quality with higher scores indicating the proper methodological quality [13]. The NOS adapted for cross-sectional studies was independently used by two researchers to quality assessment. Any probable discrepancies were resolved through discussion, with a third author involvement. The articles scoring 3–9 were included for data extraction and meta-analysis.

### Data extraction and analysis

This process was performed independently by two researchers using a pre-prepared checklist (including First author’s name, Publication year, Study location, Age group, Sample size, CIPN prevalence, and Study tool). Comprehensive Meta-Analysis software (CMA, v.2) was hired for data analysis. The Random Effect model was utilized for prevalence calculation based on the CI95%. Subgrouping analysis was also performed based on CIPN scales. The study heterogeneity was assessed using I^2^ index and publication bias was examined through the Begg and Mazumdar correlation test and Funnel plot analysis.

## Results

### Key characteristics of eligible studies

**Fig. 1:**
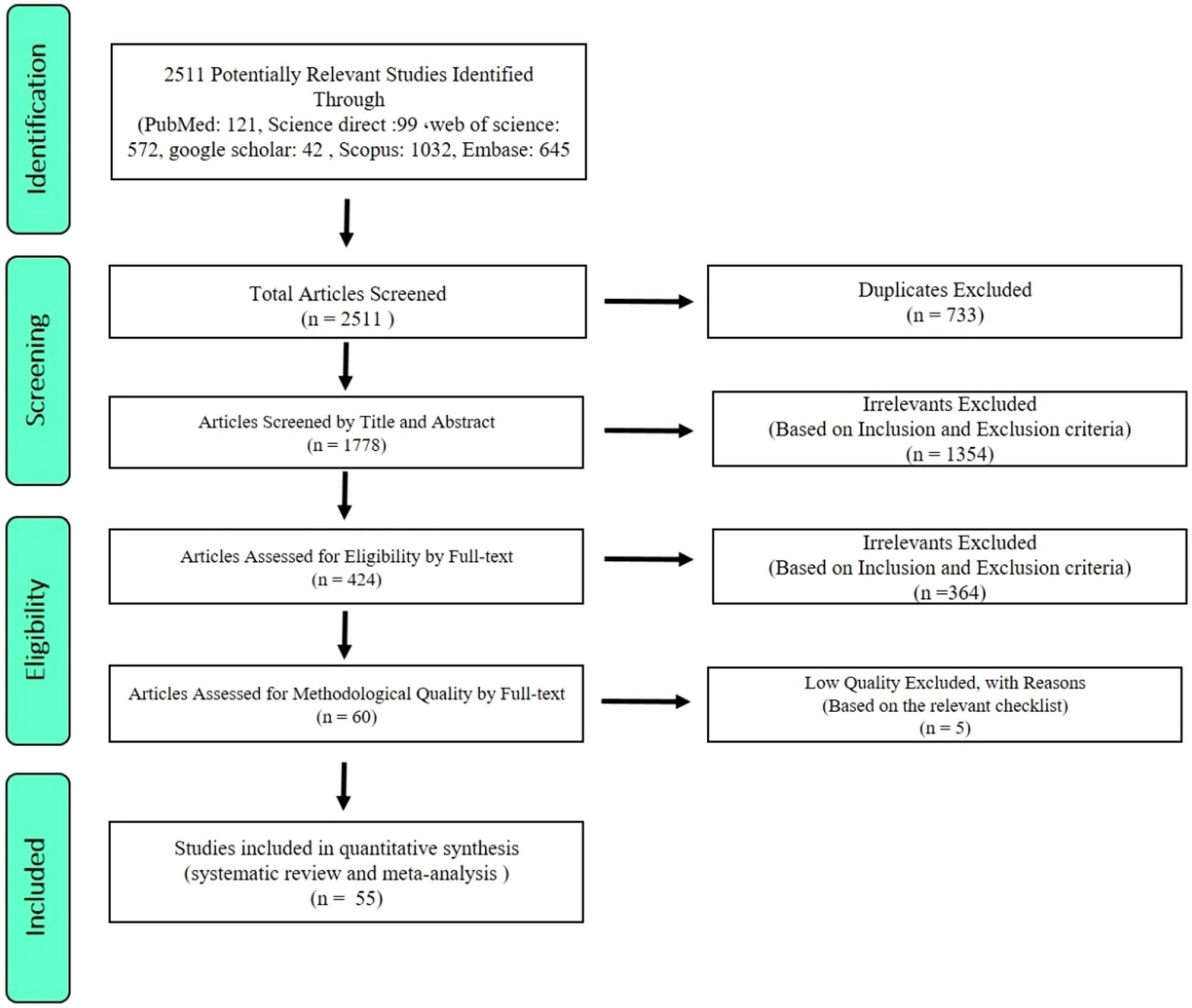
The flowchart representing the stages of included studies in systematic review and meta-analysis (PRISMA 2009)

Following searching of databases, 2511 potentially relevant articles were gathered. 733 papers were excluded due to duplication. Subsequently, Titles and Abstracts of articles were evaluated based on Inclusion/Exclusion criteria. In this primary screening process, 1354 studies were also removed. Following secondary screening process, full texts were assessed and 364 articles were excluded. 60 eligible studies were selected and underwent quality assessment using the NOS. Following exclusion of 5 poor methodological quality articles, 55 eligible studies were selected for data analysis (Fig. 1). The search process of various databases is reported in Table 1.

**Table 1:** Searching strategy for paper collection using various electronic databases

Database	Type of Searching	Search strategy	No of collected papers
PubMed	Advanced	(chemotherapy [tiab]) AND (“peripheral neuropathy” [tiab] OR “peripheral neurotoxicity” [tiab] “CIPN” [tiab] OR “peripheral nerve disease” [tiab] OR “PNS” [tiab]) AND (Occurrence [tiab] OR Burden [tiab] OR outbreak [tiab] OR prevalence [tiab] OR Epidemiology [tiab])	121
Science Direct	Advanced	(chemotherapy) AND (“peripheral neuropathy” OR “peripheral neurotoxicity” OR “CIPN” OR “peripheral nerve disease” OR “PNS”) AND (Occurrence OR Burden OR outbreak OR prevalence OR Epidemiology):	-
		(chemotherapy) AND (“peripheral neuropathy” OR “peripheral neurotoxicity” OR “CIPN” OR “peripheral nerve disease” OR “PNS”) AND (Occurrence OR Burden OR outbreak)	67
		(chemotherapy) AND (“peripheral neuropathy” OR “peripheral neurotoxicity” OR “CIPN” OR “peripheral nerve disease” OR “PNS”) AND (prevalence OR Epidemiology)	32
Scopus	Advanced	TITLE-ABS-KEY(chemotherapy) AND TITLE-ABS-KEY(“peripheral neuropathy” OR “peripheral neurotoxicity” OR “CIPN” OR “peripheral nerve disease” OR “PNS”) AND TITLE-ABS-KEY(Occurrence OR Burden OR outbreak OR prevalence OR Epidemiology)	1032
WOS	Advanced	TS=(chemotherapy) AND TS=(“peripheral neuropathy” OR “peripheral neurotoxicity” OR “CIPN” OR “peripheral nerve disease” OR “PNS”) AND TS=(Occurrence OR Burden OR outbreak OR prevalence OR Epidemiology)	572
Embase	Advanced	(chemotherapy: ti, ab, kw) AND (‘peripheral neuropathy’:ti, ab, kw OR ‘peripheral neurotoxicity’:ti, ab, kw OR ‘CIPN’:ti, ab, kw OR ' peripheral nerve disease’:ti, ab, kw OR ‘PNS’:ti, ab, kw) AND (‘Occurrence’:ti, ab, kw OR ‘Burden’:ti, ab, kw OR ‘outbreak’:ti, ab, kw OR ‘prevalence’:ti, ab, kw OR ‘Epidemiology’:ti, ab, kw)	645
Google Scholar	Advanced	allintitle: (chemotherapy) (“peripheral neuropathy” OR “peripheral neurotoxicity” OR “CIPN” OR “peripheral nerve disease” OR “PNS”) (Occurrence OR Burden OR outbreak OR prevalence OR Epidemiology)	42

## Participants

The included participants were all survivors of acute lymphoblastic leukemia, breast cancer, colorectal cancer, lymphoma, gastric cancer, multiple myeloma, ovarian cancer, testicular cancer, and lung cancer. All participants undergoing treatment with Taxanes, Platinum-based compounds, Vinca alkaloids, Bortezomib, Lenalidomide, and other neurotoxic drugs (Table 2).

### Design and settings

Cross-sectional and survey studies were incorporated in this investigation. 10studies (out of 55) were surveys, and the remaining were cross-sectional studies. These studies were conducted in 23 different countries mostly affiliated to the USA and Australia, respectively (Table 2).

**Table 2:** Summary of characteristics of included studies representing the overall prevalence of CIPN

No.	Author	Year	Country	Age range or (mean ± SD) year	Sample size	Instrument (Overall prevalence)	Instrument (motor prevalence)	Instrument (sensory prevalence)
1	Hsieh et al. [6]	2023	Taiwan	38–80	75	TNS-c (77.3%), PNQ (37.3%)	TNSc (17%), PNQ (16%)	TNSc (54.7%), PNQ (35%)
2	Ben Kridis et al. [14]	2023	Tunisia	13–80	73	DN4 questionnaire (52.1%)	-	-
3	Sheikh-Wu et al. [15]	2022	USA	21–88	117	Therapy-Related Symptom Checklist (68%)	-	-
4	Rodwin et al. [8]	2022	USA	17.1 ± 7.7	148	History + Exam (37.8%)	History + Exam (83.9%)	History + Exam (25%)
5	Nielsen et al. [7]	2021	Denmark	18–99	2839	EORTC-CIPN20(CIPN20) (17%)	-	-
6	Tunjungsari et al. [9]	2021	Indonesia	median age 7, 4–18	52	Complaints (26.9%), TNS-PV (76.9%), NCS (100%), [NCS + TNS-PV+ complaints] (25%)	NCS (100%)	NCS (76.5%)
7	Frigotto et al. [16]	2022	Brazil	-	21	DN4 (47.62%)	-	-
8	Battaglini et al. [17]	2021	Australia	58 ± 10.7	986	FACT/GOG-NTX (76.5%)	-	-
9	Brady et al. [18]	2021		54.6 ± 10.9	8890	22.7%	-	-
10	Brydøy et al. [19]	2009	Norway	median:39 (24–73)	1378	SCIN (29%)	-	-
11	Hershman et al. [20]	2010	USA	median: 51 (34–80)	50	FACT/GOG-Ntx (> 80%)	-	-
12	Webber et al. [21]	2019	Australia	median 50–60 (18- over70)	1356	FACT/GOG-Ntx (78.1%)	-	-
13	Ponce et al. [22]	2018	Spain	61.6 ± 12.6	384	DN4 (7.3%)	-	-
14	McCrary et al. [23]	2019	Australia	59 ± 13	100	EORTC CIPN-20 (87%)	-	-
15	McCrary et al. [24]	2019	Australia	57 ± 13	190	CIPN20 (67.9%)	-	-
16	Martínez et al. [25]	2019	Colombia	57 ± 13	1,551	“patients’ medical records” (49.9%)	-	-
17	Huang et al. [26]	2018	USA	31.8 ± 8.4	2811	CTCAE (9%)	-	-
18	Hong et al. [27]	2019	China	-	254	CIPNAT (74.02%)	-	-
19	Haidinger et al. [28]	2019	Germany	median: 49 ,20–81	1,122	unknown survey (65–69%)	-	-
20	Ezzi et al. [29]	2019	Kenya	median: 51, 14–80	67	TNS (83.6%)	-	-
21	Donovan et al. [30]	2019	USA	49.1 ± 11.8	3,061	2010 LIVESTRONG survey (33.7%)	-	-
22	Zaleta et al. [31]	2018	USA	55 ± 10	680	history of CIPN in a cancer registry (30%)	-	-
23	Shah et al. [32]	2018	USA	58.1 ± 16.4	509	electroic records: AAN criteria based diagnosis (52.7%)	-	-
24	Schilling et al. [33]	2018	Germany	-	1116	the NCCN distress thermometer (34%)	-	-
25	Moreira et al. [34]	2018	Brazil	45 -81	100	a form consist of CTCAE4&the neurological examination (56%)	-	-
26	Magee et al. [35]	2018	UK	-	601	DN4 (18.8%)	-	-
27	Kandula et al. [36]	2018	Australia	median 16(7–47)	100	ped-mTNS (53%)	-	-
28	Vasquez et al. [37]	2012	Ireland	-	29	mTNS (93%)	-	-
29	Jain et al. [38]	2014	India	5–18	80	rTNS (33.75%)	-	-
30	Vasquez et al. [39]	2013	Ireland	median : 62, 31–74	29	mTNS (100%)	-	-
31	Liew et al. [40]	2013	Canada	median 41.0, 21.5–71.9	29	SPNS (43%)	-	-
32	Burnette et al. [41]	2013	USA	18- >80y	736	researcher made (27%)	-	-
33	Boland et al. [42]	2013	UK	median : 60, 41–71	32	s-LANSS (50%)	-	-
34	Beijers et al. [43]	2015	the Netherlands	-	130	EORTC QLQ-CIPN20 (54%)	-	-
35	Padman et al. [44]	2014	Australia	Median 66, Range 45–79	25	EORTC QLQ-CIPN20 (72%)	-	-
36	Simon et al. [45]	2017	USA	56.7 ± 11.8	126	QLQ-CIPN20 (73%)	-	-
37	Webber et al. [46]	2015	Australia, the UK, USA and Canada	≥ 18	1085	online survey (78.4%)	-	-
38	Ahn et al. [47]	2016	Korea	-	478	55.2%	-	-
39	Beijers et al. [48]	2015	The Netherlands	67.5 ±9.3	156	ICPNQ (65%), EORTC QLQ-CIPN20 (53%)	-	-
40	Imam et al. [49]	2016	SUDAN	18–65	78	WHO grading scale (47.5%)	-	-
41	Ali et al. [50]	2017	USA	median: 60, 31–93	605	Medical records (26.8%)	-	-
42	Kandula et al. [51]	2017	Australia	7 to 47	110	NCS and novel nerve excitability studies (33%)	-	-
43	Tay et al. [52]	2017	Malaysia	4.8–18.0	101	cTNS (26.7%)	-	-
44	Timmins et al. [53]	2020	Australia	44–85	47	FACT/GOG-Ntx13 (80.9%)	-	-
45	Ramchandren et al. [54]	2009	USA	14.4 ± 2.8	37	NCS (29.7%)	-	-
46	Mizrahi et al. [4]	2022	Australia	median:58.0	252	FACT/GOG-Ntx-13 (66%)	-	-
47	Kautio et al. [55]	2010	Finland	55.8 ± 8.6	152	NCI-CTC (59%)	-	-
48	Beijers et al. [56]	2014	The Netherlands	60.7 ± 11	43	FACT/GOG-Ntx (most of the patients experienced neurotoxicity in the upper and lower extremities 78.8% and 89.7%, respectively)	-	-
49	Zaleta et al. [57]	2020	USA	62.6 ± 9	289	PROMIS-29 (21%)	-	-
50	Hung et al. [5]	2021	Taiwan	27–89	93	-	NCI-CTCAE 4.03v (47.3%)	NCI-CTCAE4.03v (53.8%)
51	Vivas-Rosales et al. [58]	2017	Mexico	9.7 ± 3.1	32	-	ped-mTNS (34.3%)	ped-mTNS (78.1%)
52	Selvy et al. [59]	2021	France	66.7 ± 10.4	67	-	-	QLQ-CIPN20 (26.9%)
53	Selvy et al. [60]	2020	France	31.1–89.3	406	-	-	EORTC QLQ-CIPN20 (31.3%)
54	Bonhof et al. [10]	2020	The Netherlands	painful:65.9 ± 8.9/ non-painful:66.1 ± 8.6	477	-	-	EORTC QLQ-CIPN20 (31.027%)
55	Beijers et al. [61]	2014	The Netherlands	-	188	-	-	EORTC QLQ-CIPN20 (71.27%)

### Types of CIPN

**Fig. 2:**
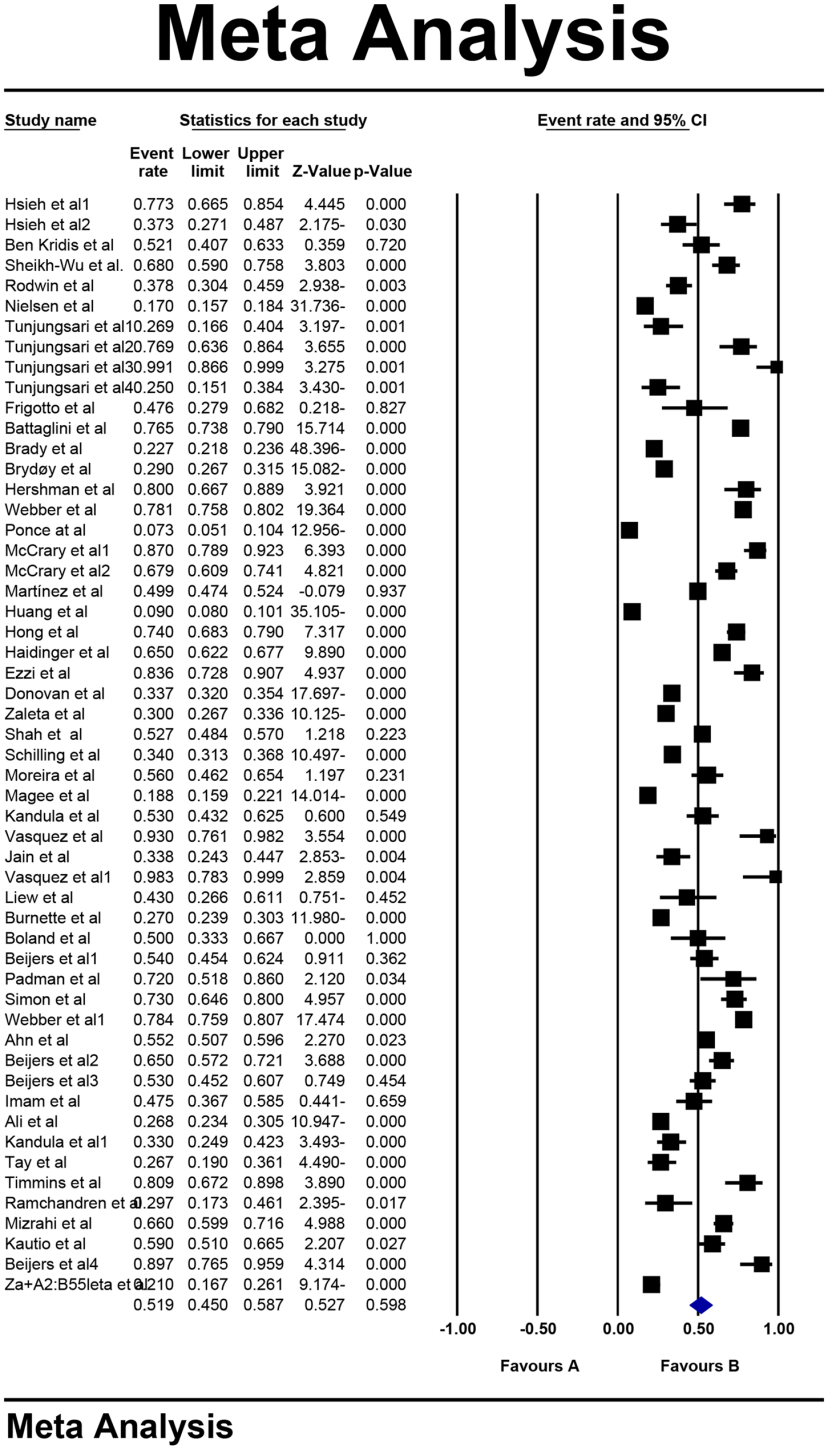
Overall prevalence of CIPN representing forest plot based on random effect method

**Fig. 3:**
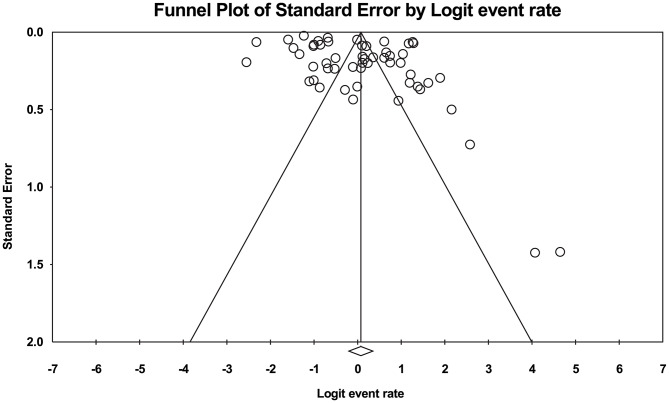
Funnel plot diagram representing distribution bias in reviewed studies

**Fig. 4:**
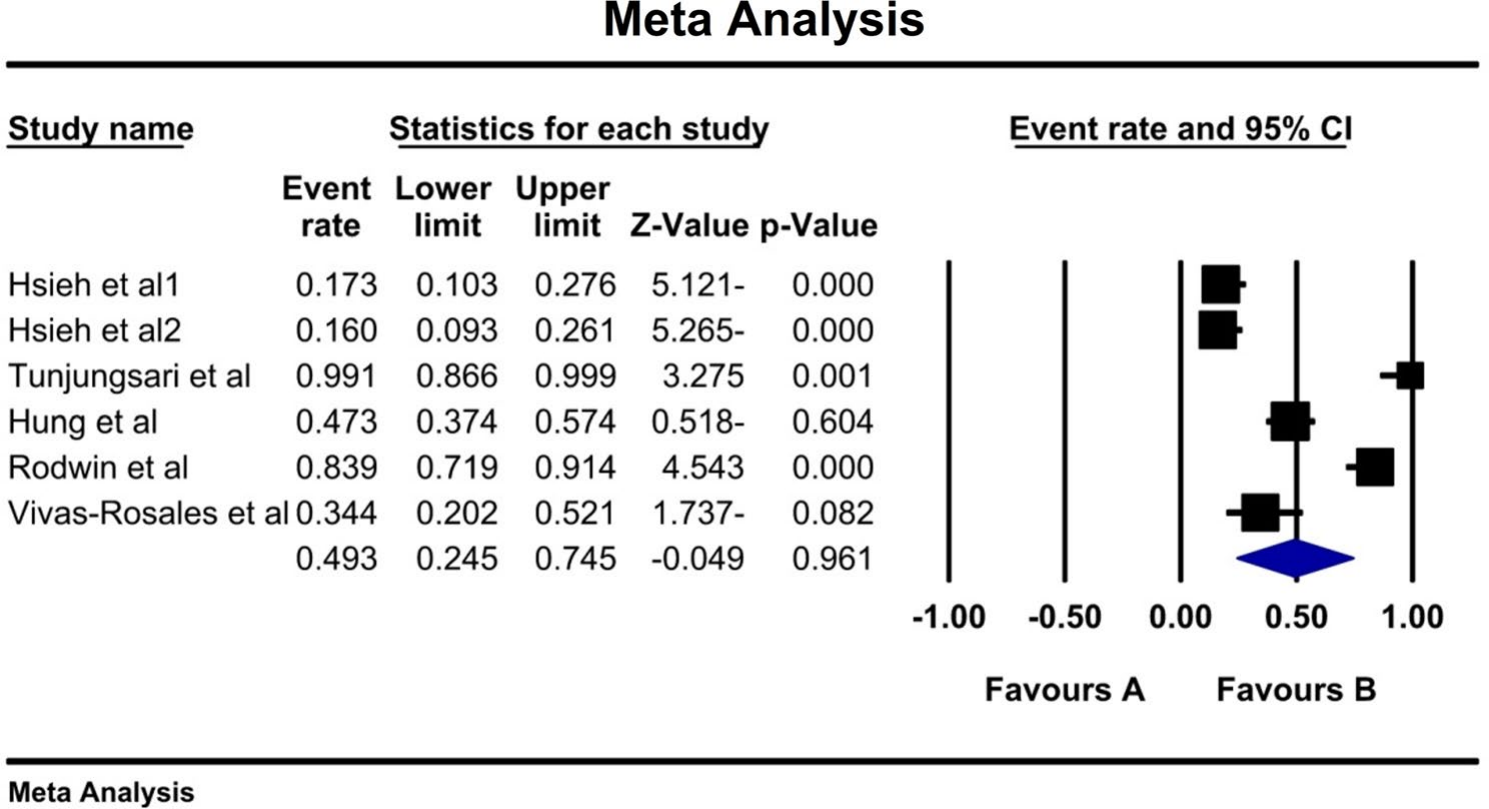
Overall forest diagram of motor CIPN based on random effect method

**Fig. 5:**
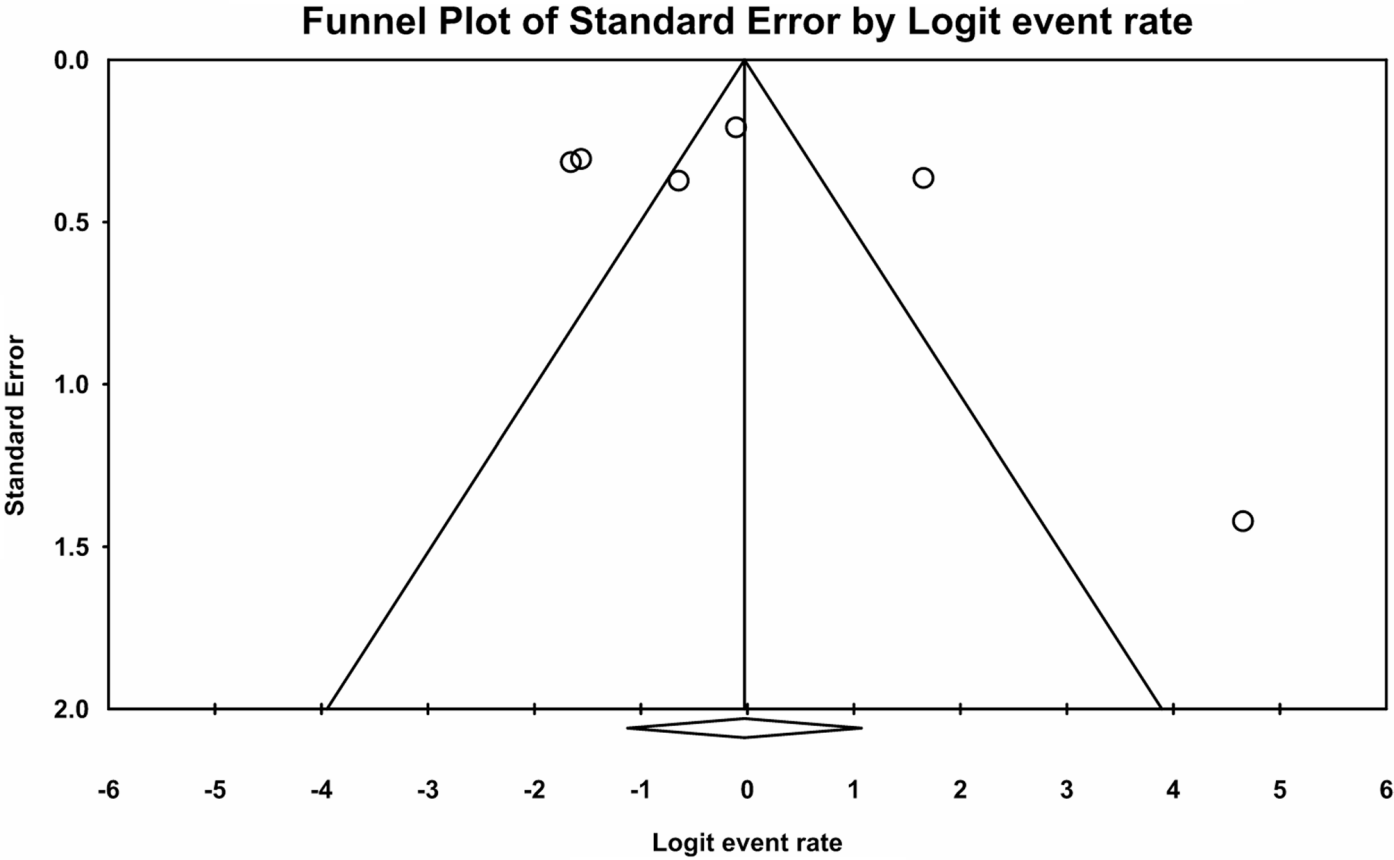
Funnel plot diagram of distribution bias in reviewed studies

**Fig. 6:**
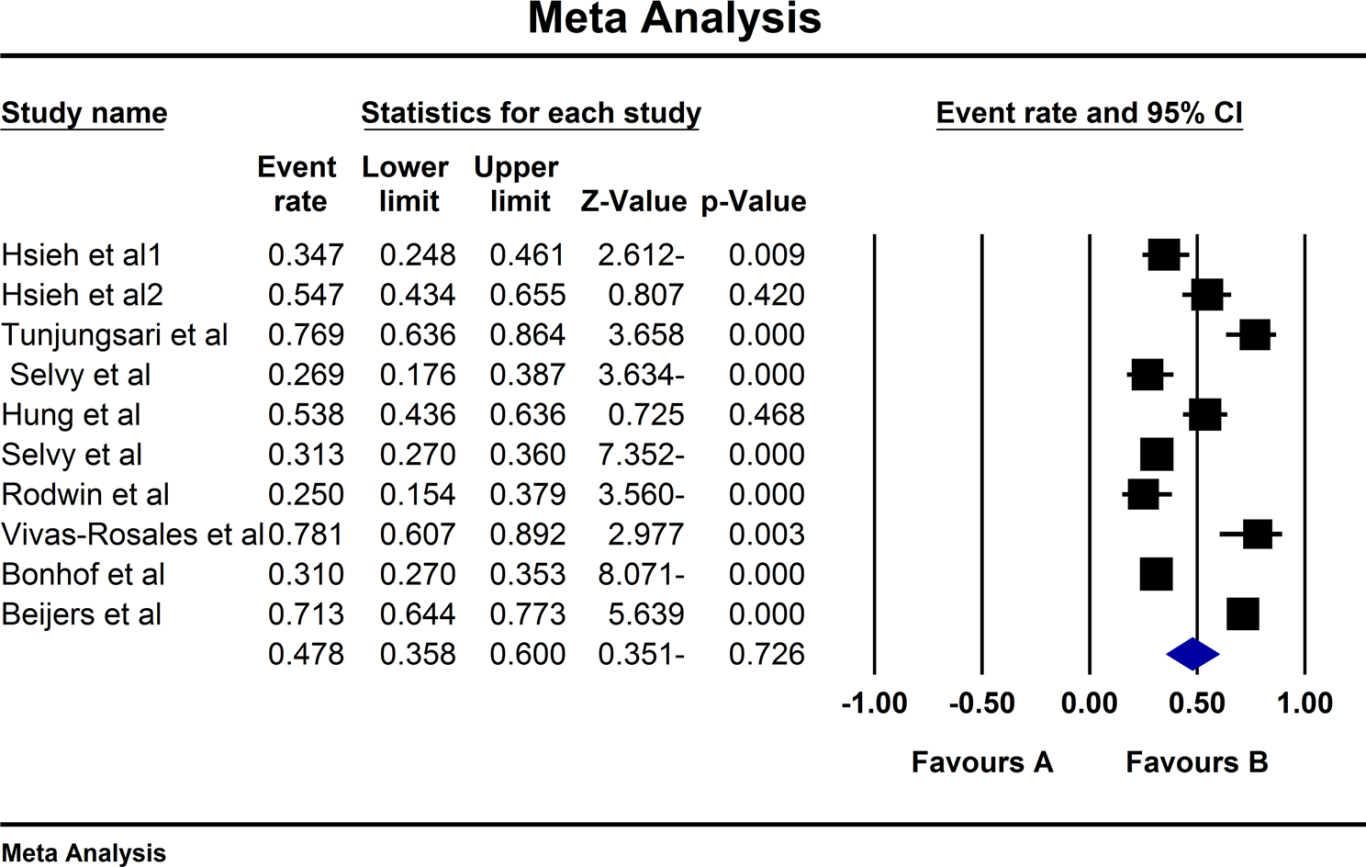
Overall forest plot diagram of sensoy CIPN based on random effect method

**Fig. 7:**
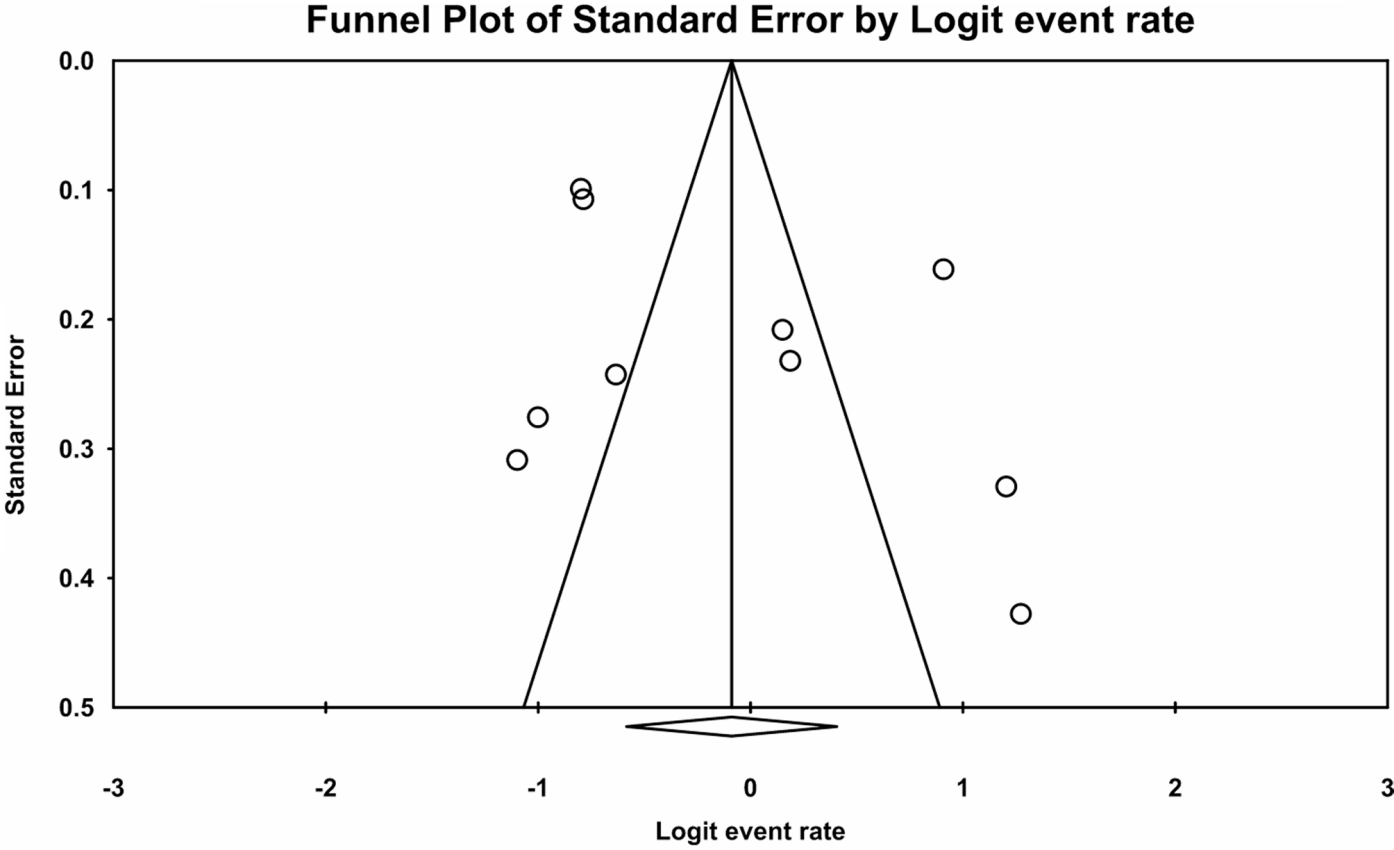
Funnel plot diagram of distribution bias in reviewed studies

49 studies reported general CIPN, 5 studies focused on motor CIPN, 9 studies examined sensory CIPN, and 1 study reported Autonomic CIPN. Additionally, in the studies conducted by Hsieh et al. (2023), Tunjungsari et al. (2021), and Beijers et al. (2015), prevalence was reported due to the application of various assessment tools. Thus, separate statistical analyses were conducted and reported for Overall, Motor, and Sensory CIPN values (Figs. 2, 3, 4, 5, 6 and 7).

### Overall CIPN

Following the assessment of 49 studies, 54 types of CIPN prevalence were reported (n:33,667 individuals). High levels of heterogeneity (I^2^:99.04%) were found and Random Effect model was utilized. Consequently, based on the meta-analysis investigations, the overall prevalence of CIPN was reported 51.9% (95% CI:45-58.7) (Fig. 2). Furthermore, the Begg and Mazumdar correlation test indicated no publication bias (p:0.899) (Fig. 3).

### Motor CIPN

Following the assessment of 5 studies, a total of 6 types of prevalence were reported (n:383 individuals). High heterogeneity (I^2^:93.5%) was reported leading to the utilization of a Random Effect model. Consequently, the overall prevalence of motor CIPN was found 49.3% (95% CI:24.5–74.5) (Fig. 4). Furthermore, Begg and Mazumdar correlation test indicated no publication bias (p:0.707) (Fig. 5).

### Sensory CIPN

Following the assessment of 9 studies, a total of 10 types of prevalence were reported (n:1521 individuals). High heterogeneity (I^2^:94.3) was reported leading to the utilization of Random Effect model. Consequently, the overall prevalence of sensory CIPN was reported 47.8% (95% CI:35.8–60) (Fig. 6). The Begg and Mazumdar correlation test indicated no publication bias (p:0.474) (Fig. 7).

### Subgroup analysis of scales

According to the various utilized tools, the highest reported prevalence of CIPN was associated with Composite Scales with a prevalence of 69.6% (95% CI:50–84) (Table 3).

**Table 3:** Subgrouping analysis based on the scales

	Scale	N	Sample size	I^2^	Begg and Mazumdar correlation test	Prevalence (95% CI)
CIPN	Common Toxicity Criteria	3	3041	99.1	1.000	33.4 (95%CI: 6.5–78.4)
	Composite Scales	8	533	93.1	0.265	69.6 (95%CI: 50–84)
	Patient-Reported Outcomes	22	9666	99.06	0.693	56 (95%CI: 42.8–68.3)
	Pain Scales	4	1079	96.3	0.734	26.1 (95%CI: 11-50.3)
	Nerve Conduction Studies	3	199	86.06	1.000	51 (95%CI: 21.7–79.6)
	Others	15	19,186	99.3	1.000	43.5 (95%CI: 33.4–54.2)

## Discussion

The aim of this systematic review and meta-analysis study is to estimate the global prevalence of CIPN in cancer patients. CIPN is a common complication in cancer patients undergoing treatment with neurotoxic chemotherapy drugs. The prevalence of CIPN is increasing with the growing number of cancer survivors [62]. According to our findings, the prevalence of overall CIPN, Motor CIPN, and Sensory CIPN were found 51.9%, 49.3%, and 47.8%, respectively.

In a systematic review and meta-analysis study conducted by Seretny and colleagues (2014), a prevalence of CIPN of 30% following 6 months post-chemotherapy completion reported [11]. In contrast, our study estimated the long term CIPN prevalence at 51.9%. The findings of the present study were 1.5 times higher than the prevalence reported by Seretny in 2014. This variation was probably associated with the number or design of studies.

In various investigations, the prevalence of CIPN has been reported from 7.3% [22] to 100% [39]. This variability in CIPN prevalence can be attributed to the treatment regimens. Nielsen et al. (2021) reported the prevalence of CIPN at 17% with a sample size of 2,839 individuals [7]. Besides, in their study, the prevalence of CIPN was estimated in the general population of oncology-based patients with various disease diagnoses and treatment regimens. Basically, this value seems to be lower than the situations receiving neurotoxic chemotherapy drugs. Conversely, the prevalence of CIPN in studies conducted by Ezzi et al. (2019) resulting from Cisplatin administration and Timmins et al. (2020) resulting from Taxane administration were 83.6% and 81%, respectively [29, 53]. Among neurotoxic chemotherapy drugs, Platinum-based agents, Taxanes, Ixabepilone, and Thalidomide have been associated with the highest toxicity levels [3].

Furthermore, the diversity in reported prevalence of CIPN is probably associated to the tools used for CIPN measurement. In the study conducted by Tunjungsari et al. (2021), the CIPN prevalence was calculated using 4 different methods resulting in four different prevalence rates (*n* = 52 individuals) [9]. Accordingly, subgrouping analysis was conducted in the current study based on the type of tools or the scales. The findings indicated that the highest prevalence of CIPN based on the Composite Scales was 69.6%, while the lowest reported prevalence was related to the Pain Scales at 26.1%. There are various types of Composite Scales including the Total Neuropathy Score Scale (TNS), as a valid clinician-based tool, that is potentially capable of identifying mild changes in sensory CIPN in 77% of cases. On the other hand, despite the application of pain assessment tools in CIPN evaluation, most of these tools are neither specific nor sufficiently valid for measurement of CIPN. In a review study conducted by Park et al. (2019), the Patient-Reported Outcome (PRO) tool and Composite scales were introduced as valid tools, while pain assessment tools were considered non-specific [63]. In the present study, the results of subgrouping analysis also supported the findings of Park and colleagues.

Furthermore, our study findings regarding the high prevalence of CIPN and the diversity of assessment tools align with the systematic review and meta-analysis study conducted by Teng et al. (2021) concerning the Oxaliplatin-induced CIPN [64].

As highlighted in various studies, the diversity and multitude of CIPN assessment tools, along with the absence of a standardized tool, lead to different prevalence rates and ambiguity in current understanding of this condition, posing challenges for health policy decisions [63, 64]. Thus, it is recommended that international organizations, including the WHO and active international associations in oncology and neurology, introduce a standardized tool for CIPN measurement in the form of clinical guidelines. These guidelines standardize the practices in this field. Additionally, screening of patients for CIPN at multiple time points (including pre-chemotherapy, intra-procedural chemotherapy, post-chemotherapy, and during follow-up periods) can aid in diagnosis and management of acute and chronic phases of CIPN. Documentation of CIPN screenings at various time points of medical records (using a standardized tool) can facilitate future research. This guideline can potentially lead to assessment of the current status based on more detailed and accurate information, considering the time effect.

### Limitation

The most important limitation of the present study was that the studies reviewed homogeneously based on cancer type and chemotherapy regimen did not report the prevalence of CIPN to be used in the analysis. Also, due to the heterogeneous geographical distribution, analysis by continent was not possible.

## Conclusion

The global prevalence of CIPN in a heterogeneous population of cancer patients is approximately 50%. Also, based on the different instruments used, the highest reported prevalence of CIPN with a prevalence of 69.6% was related to the composite measures. According to the remarkable advancements in cancer treatment and enhancement in the number of survivors, management of CIPN is a considerable concern to preserve the quality of life of survivors and reduce the associated treatment costs imposed on patients and the healthcare system. Integration of necessary strategies for prevention, screening, and treatment of CIPN into clinical guidelines seems a critical and strategic approach for CIPN management.

## Data Availability

Datasets are available through the corresponding author upon reasonable request.
